# Prognostic accuracy of Neonatal SOFA score versus SIRS criteria in preterm infants with late-onset sepsis

**DOI:** 10.1007/s00431-023-05143-5

**Published:** 2023-08-12

**Authors:** Chiara Poggi, Martina Ciarcià, Francesca Miselli, Carlo Dani

**Affiliations:** 1 Neonatal Intensive Care Unit, Department of Mother and Child Care, Careggi University Hospital, Florence, Italy; 2 Department of Neurosciences, Drug Research and Child Health, University of Florence, PsychologyFlorence, Italy; 3 Department of Clinical and Experimental Medicine, University of Modena and Reggio Emilia, Modena, Italy

**Keywords:** Newborn, Preterm, Late onset sepsis, nSOFA, SIRS, Mortality

## Abstract

Neonatal SOFA score was reported as an accurate predictor of mortality while the prognostic accuracy of SIRS criteria is unknown. The aim was to compare neonatal SOFA and SIRS criteria for the prediction of late onset sepsis-related mortality in preterm newborns. Newborns ≤ 32 weeks with late onset sepsis were retrospectively studied. Neonatal SOFA and SIRS criteria were calculated at onset of sepsis (T0), and after 6 ± 1 (T1), 12 ± 3 (T2) and 24 ± 3 h (T3). Outcome was death during antibiotic treatment for late onset sepsis. We studied 112 newborns with gestational age 26.9 ± 2.3 weeks; 11% met the study outcome. Neonatal SOFA was significantly higher in non-survivors vs. survivors at all time intervals; SIRS criteria were significantly higher in non-survivors vs. survivors at T1, T2 and T3. Neonatal SOFA increased over time in non-survivors (p = 0.003). At T0, the area under receiver operating characteristics curve was significantly higher for neonatal SOFA score than SIRS criteria (0.950 vs. 0.569; p = 0.0002), and the best calculated cut-off for T0 neonatal SOFA score was 4. In multivariate analysis T0 and T1 neonatal SOFA were predictors of late onset sepsis-related mortality (p = 0.048 and p < 0.001).

*Conclusion:* Neonatal SOFA score showed greater discriminatory capacity for mortality than SIRS criteria and might be helpful to plan management for patients at higher risk of death.

**Table:** 

**What is Known:** *• Neonatal SOFA score may be an accurate prognostic tool.* *• No prognostic score has been fully standardized for septic newborns in NICU.*	
**What is New:** *• Neonatal SOFA score outperformed SIRS criteria for the prediction of prognosis in preterm infants with late onset sepsis.* *• Neonatal SOFA score assessed at onset of sepsis and 6 hrs later is a predictor of mortality.*

**Supplementary Information:**

The online version contains supplementary material available at 10.1007/s00431-023-05143-5.

## Introduction

Late onset sepsis (LOS) is a major cause of morbidity for preterm infants in NICU, affecting 10–30% of very low birthweight infants [1–3] with a mortality rate of 7–15% [4, 5]. According to Sepsis-3 consensus, sepsis definition in adults is centered on organ dysfunction due to dysregulated host response to infection [6] while the definition of sepsis in newborns is still based on Systemic Inflammatory Response Syndrome (SIRS) criteria alongside with the presence of infection, as proposed by the International Pediatric Sepsis Consensus Conference in 2005 [7]. Prognostic assessment of critically ill septic adults is based on Sequential Organ Failure Assessment (SOFA) score [6] while no scoring system has been fully standardized for newborns in NICU. Neonatal SOFA (nSOFA) was developed as a scoring system for organ dysfunction in preterm infants with LOS [8] and showed valuable prognostic accuracy in newborns with gestational age < 33 weeks [5, 8, 9]. Moreover, the progression of organ failure in preterm infants who die because of LOS showed a definite temporal relationship with death [10].

Although SIRS criteria are diagnostic rather than prognostic tools, both in adults and pediatrics they were compared to SOFA score for the prediction of sepsis-related mortality [11, 12]. A significantly higher prognostic capacity for sepsis-related mortality and morbidity was reported for SOFA score than SIRS criteria [11, 12], showing that organ impairments rather than signs of inflammation are the key elements for prognostic assessment of patients with sepsis. The prognostic capacity of neonatal SIRS criteria is currently unknown. On these bases we hypothesized that nSOFA is a better prognostic marker of mortality than SIRS criteria in preterm newborns with LOS. Thus, the aim of this study was to compare the accuracy of nSOFA score with SIRS criteria for the prediction of LOS-related mortality in preterm newborns.

## Methods

### Study design and participants

This retrospective single center study was approved by the pediatric local ethics committee. Preterm infants who were born at ≤ 32 weeks gestational age from January 2016 to December 2021 and experienced an episode of LOS during NICU stay at Careggi University Hospital, Florence, Italy, were enrolled in the study. Exclusion criteria were the presence of major congenital abnormalities or genetic syndromes and inborn errors of metabolism. LOS was defined as positive blood culture taken after the first 72 h of life [13]. In order to exclude contaminated samples, in cases of blood culture growing coagulase negative Staphylococcus species, patients were considered as having LOS only if C-reactive protein (CRP) was > 10 mg/L and they received antibiotics for > 5 days [14, 15]. Blood samples for cultures were obtained from peripheral vein (at least 1 mL) [16] with strict adherence to the sterile technique and collected in dedicated vials (BD Bactec^TM^, Becton Dickinson and Company, Sparks, USA). The primary outcome of the study was the comparison of accuracy of nSOFA and SIRS criteria in predicting LOS-related mortality defined as death occurring during ongoing antibiotic treatment for LOS.

At onset of sepsis (T_0_) each enrolled patient was sampled for blood culture, complete blood count, CRP and procalcitonin (PCT). Neonatal SOFA score and SIRS criteria were calculated at T_0_, and after 6 ± 1 (T_1_), 12 ± 3 (T_2_), and 24 ± 3 h (T_3_). As per local protocol, cases of LOS received empiric treatment with vancomycin and amikacin or other aminoglycoside; if a previous course of antibiotics had been administered within 7 days before the onset of LOS, different antibiotic regimens including carbapenem and second line anti-staphylococcal drugs were considered. Targeted antibiotic treatment was based on sensitivity of the isolates. All cases of LOS were treated with antibiotics for at least 5 days or until death.

Inotropic drugs (i.e. adrenaline, noradrenaline, dopamine, dobutamine, etc.) and glucocorticoids for cardiovascular impairment were administered and titrated consistently with the findings of functional echocardiography and/or monitoring of systemic arterial pressure and lactate levels, according to the American College of Critical Care guidelines for the treatment of neonatal shock [17]. Concomitant treatments, such as sedatives, analgesics, caffeine, ibuprofen and paracetamol for the treatment of patent ductus arteriosus, steroids for purposes other than increasing BP, and parenteral nutrition were administered according to local protocols.

Infants were started on mechanical ventilation when the pH was < 7.20 with PaCO2 > 65 mm Hg, or PaO_2_ < 50 mmHg with FIO_2_ ≥ 0.50, after surfactant treatment, or if infants had frequent episodes of apnea. Mechanical ventilation was set to maintain a PaCO_2_ of 55 to 65 mmHg and 90–95% pulse oxygen saturation (SpO_2_). All data were extracted from local electronic clinical charts.

### SIRS criteria and nSOFA assessment

Neonatal SOFA score (score 0–15) was calculated taking into account respiratory, cardiovascular and hematologic sub-scores, as previously reported [5, 8]. Categorical scores were assessed for each of the following: (a) need for mechanical ventilation and oxygen requirement during mechanical ventilation (score 0–8: 0, not intubated or intubated and SpO_2_/FiO_2_ ≥ 300; 2, intubated and SpO_2_/FiO_2_ < 300; 4, intubated and SpO_2_/FiO_2_ < 200; 6, intubated and SpO_2_/FiO_2_ < 150; 8, intubated and SpO_2_/FiO_2_ < 100); (b) administration of inotropes or glucocorticoids for cardiovascular impairment (score 0–4; 0, no inotropes and no steroids; 1, steroids, no inotropes; 2, one inotrope, no steroids; 3, ≥ 2 inotropes, no steroids or 1 inotrope and steroids; 4, ≥ 2 inotropes and steroids); (c) most recent platelet count (score 0–3; 0, > 150 × 10^3^/mm^3^; 1, 100–149 × 10^3^/mm^3^; 2, < 100 × 10^3^/mm^3^; 3, < 50 × 10^3^/mm^3^).

SIRS criteria (score 0–4) were calculated according to the International Pediatric Sepsis Consensus neonatal definition [7] as follows: (a) abnormal body temperature, < 36 °C or > 38.5 °C; (b) abnormal heart rate, tachycardia > 180 bpm or bradycardia < 100 bpm for at least 30 min; (c) respiratory distress, respiratory rate > 60/min or need for mechanical ventilation; (d) hematologic impairment, white blood cells > 15 × 10^3^/mm^3^ or < 5 × 10^3^/mm^3^.

A priori rule was established for calculating nSOFA score or the number of SIRS criteria in the event of death during the assessment period. This approach is strongly recommended for studies on adult SOFA score to avoid missing data for patients with potentially high scores, in order to prevent a survivorship bias with paradoxical underestimation of the score for patients experiencing death during the assessment period [18]. To date, no consensus exists about the most appropriate method of handling missing data due to early mortality [18]. Among the proposed strategies [18] we decided that, in case of death within the first 24 h of onset, the highest recorded value of nSOFA and SIRS criteria would be imputed for the time points following death. We chose this approach because no method considering specific extra penalty for death has been explored to date for newborns and, on the other hand, considering the last recorded instead of the highest value would not account for mortality.

### Statistical analysis

The clinical characteristics of enrolled patients were described as mean and SD for continuous parametric variables, median and interquartile range for non-parametric variables, and counts and percentage for discrete variables. Comparisons between groups were performed with Student t test for parametric continuous variables, Mann–Whitney U test for continuous nonparametric variables, such as nSOFA and SIRS criteria, and Chi-squared test for categorical variables. Changes over time of nSOFA score and SIRS criteria within the single groups were analyzed with Friedman test for repeated measures. With the purpose of measuring the discrimination performance of T_0_ nSOFA and T_0_ SIRS criteria, the receiver operating characteristic curves (ROC) for each score were analyzed to calculate the area under the curve (AUC) and the best cut-off level. The comparison between the AUC of T_0_ nSOFA score and T_0_ SIRS criteria was performed using the De Long method [19]. Variables with P < 0.05 were considered for inclusion in multivariate analysis.

Sample size was calculated assuming an AUC of 0.88 for T_0_ nSOFA score [5] and LOS-related mortality of 10% [4, 5]. In order to detect a difference in AUC between nSOFA and SIRS criteria of 20%, with alpha error = 0.05 and power of 0.80, the calculated sample size was 101.

Data were analyzed with SPSS, version 26.0 (IBM, New York, US).

This study followed the “Strengthening the Reporting of Observational Studies in Epidemiology” (STROBE) guidelines for reporting observational studies.

## Results

We studied 112 newborns with LOS, with gestational age of 26.9 ± 2.3 weeks and birth weight of 839 ± 246 g; 12/112 (11%) died because of LOS (Table 1). Death occurred between T_1_ and T_2_ in one patient (8%), and between T_2_ and T_3_ in 3/12 (25%) patients, while 8/12 patients died after completion of the assessment period. Non-survivors showed lower birth weight (674 ± 222 vs. 855 ± 244 g; p = 0.008) and gestational age (25 ± 1.6 vs. 27 ± 2.3 weeks, p = 0.002) than survivors, alongside with lower post-conceptional age (26.2 ± 1.6 vs. 29.2 ± 3.1 weeks; p = 0.001) and weight (781 ± 205 vs. 1030 ± 413 g; p = 0.049) at onset of LOS (Table 1). A higher proportion of Gram-negative strains were found in blood culture from non-survivors in comparison to survivors (67 vs. 13%, p = 0.0001), while peak CRP [85(32–116) vs. 72(25–118) mg/L; p = 0.952] and PCT [19(3–67) vs. 7(3–31) ng/mL; p = 0.105] did not differ between the two groups (Table 1).

**Table 1:** Characteristics of patients and LOS episodes

	All	Survivors	Non-survivors	P*
	N=112	N=100	N=12	
General characteristics				
Gestational age, wks	26.9 ± 2.3	27.0 ± 2.3	25.0 ± 1.6	0.002
Birth weight, g	839 ± 246	855 ± 244	674 ± 222	0.008
Apgar score 5 min	8 (7-8)	8 (7-8)	7 (6-8)	0.011
Female gender	48 (43)	40 (40)	8 (66)	0.054
Cesarean delivery	64 (57)	59 (59)	5 (42)	0.127
Antenatal steroids	98 (87)	87 (87)	11 (92)	0.355
Surfactant	95 (85)	83 (83)	12 (100)	0.123
Maximal respiratory support before LOS				
None	1 (1)	1 (1)	0 (0)	0.893
Non invasive	56 (50)	54 (54)	2 (17)	
MV (PTV/HFOV)	55 (49)	45 (45)	10 (83)	
PDA	87 (78)	75 (75)	12 (100)	0.04
NEC	3 (3)	2 (2)	1 (8)	0.26
IVH ≥ grade 3	14 (12)	8 (8)	6 (50)	0.001
PVL	9 (8)	9 (9)	n.a.	n.a.
BPD	49 (44)	49 (49)	n.a.	n.a.
ROP requiring treatment	3 (3)	3 (3)	n.a.	n.a.
Hospital stay, d	79 ± 43	85 ± 40	19 ± 13	<0.0001
Death				
Overall	16 (14)	4 (4)	12 (100)	<0.0001
Sepsis-related	12 (11)	0 (0)	12 (100)	<0.0001
Characteristics of LOS				
Days of life at onset	10 (8-17)	11 (8-17)	9 (7-18)	0.312
Weight at onset, g	994 ± 403	1030 ± 413	781 ± 205	0.049
Post-conceptional age at onset, wks	28.6 ± 3.1	29.2 ± 3.1	26.2 ± 1.6	0.001
Pathogens				
Gram positives	91 (81)	87 (87)	4 (33)	0.0001
Gram negatives	21 (19)	13 (13)	8 (67)	0.0001
Fungi	0 (0)	0 (0)	0 (0)	n.a.
Inotropes				
At least 1 drug	23 (20)	11 (11)	12 (100)	<0.0001
≥ 2 drugs	10 (9)	2 (2)	8 (67)	<0.0001
Oliguria/anuria	18 (16)	8 (8)	10 (85)	<0.0001
Maximal respiratory support during LOS				
None	0 (0)	0 (0)	0 (0)	n.a.
Non invasive	21 (19)	21 (21)	0 (0)	0.071
MV (PTV/HFOV)	91 (81)	79 (79)	12 (100)	0.071
Peak CRP (mg/L)	73 (25-117)	72 (25-118)	85 (32-116)	0.952
Peak PCT (ng/mL)	7 (3-33)	7 (3-31)	19 (3-67)	0.105
Targeted antibiotic				
Vancomycin	70 (63)	67 (67)	3 (25)	0.005
Amikacin	12 (11)	8 (8)	4 (33)	0.021
Linezolid	13 (12)	13 (13)	0 (0)	0.209
Meropenem	8 (7)	4 (4)	4 (33)	0.004
Others	9 (8)	8 (8)	1 (8)	0.406

*BPD* Bronchopulmonary Dysplasia, *HFOV* High Frequency Oscillatory Ventilation, *IVH* Intraventricular Hemorrhage, *MV* Mechanical Ventilation, *NEC* Necrotizing Enterocolitis, *PDA* Patent Ductus Arteriosus, *PTV* Patient-triggered Ventilation, *PVL* Periventricular Leukomalacia, *ROP* Retinopathy of Prematurity

*Survivors vs. Non-survivors

**Fig. 1:**
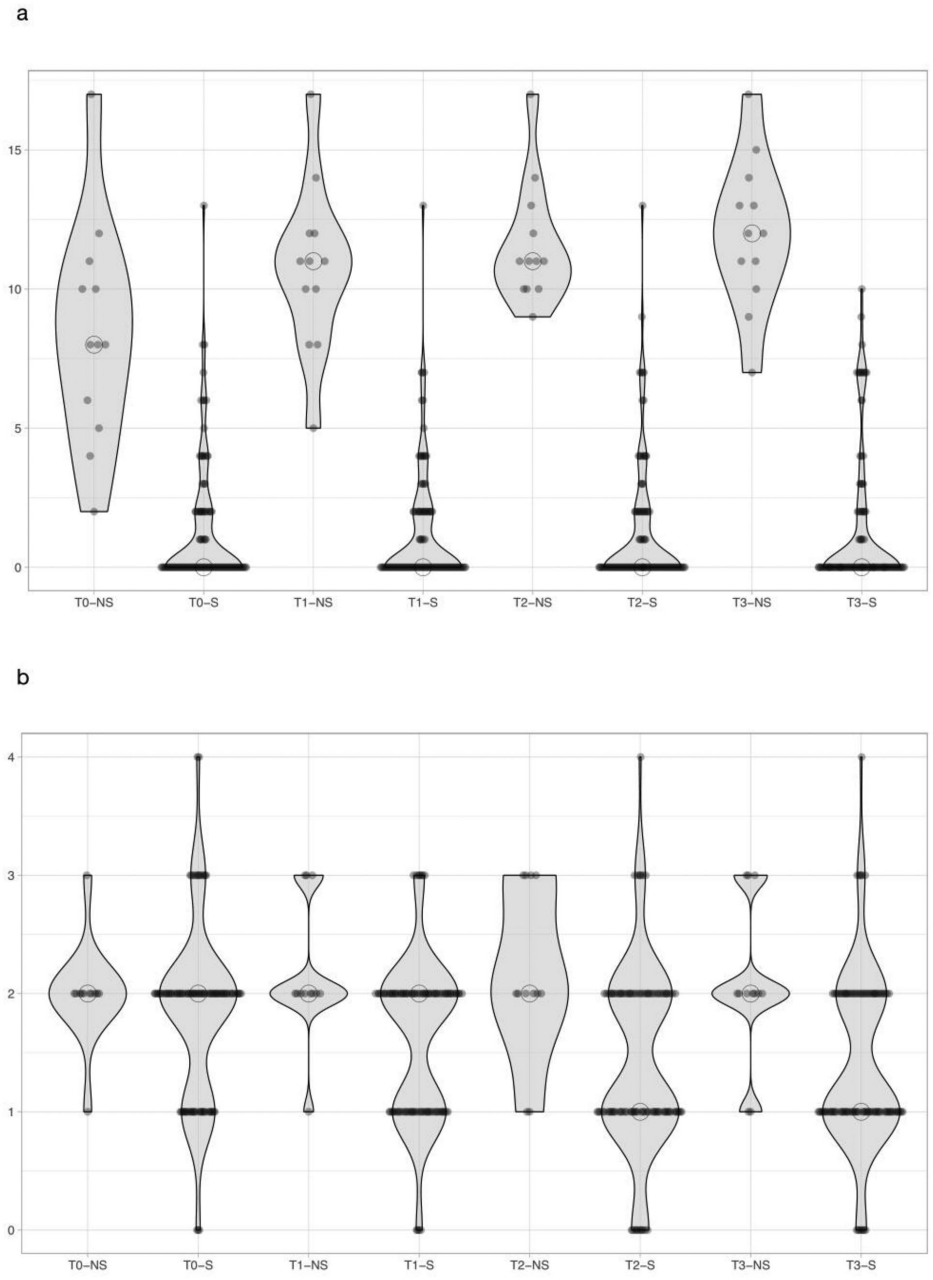
**a** Violin plot of nSOFA score at T_0_, T_1_, T_2_ and T_3_ for survivors (S) and non-survivors (NS). **b** Violin plot of SIRS criteria at T_0_, T_1_, T_2_ and T_3_ for survivors (S) and non-survivors (NS)

Neonatal SOFA score was significantly higher in non-survivors vs. survivors at T_0_, T_1_, T_2_, and T_3_ [Table 2 and Fig. 1a]. SIRS criteria were significantly higher in non-survivors vs. survivors at T_1_, T_2_, and T_3_ but were similar at T_0_ (Table 2 and Fig. 1b). Neonatal SOFA score increased during the first 24 h from onset of LOS in non survivors (p = 0.003) while it did not vary in survivors (p = 0.921); SIRS criteria did not change over time both in non survivors (p = 0.908) and survivors (p = 0.712) (Table 2).

**Table 2:** Comparison of nSOFA score and SIRS criteria between survivors and non-survivors and variations over time within survivors and non-survivors

	**T**_**0**_	**T**_**1**_	**T**_**2**_	**T**_**3**_	
	**n-SOFA score**				
Survivors, n = 100	0 (0–2)	0 (0–2)	0 (0–2)	0 (0–2)	p = 0.921
Non survivors, n = 12	8 (5–11)	11 (9–12)	11 (10–13)	12 (10–14)	p = 0.003
	p < 0.00001	p < 0.00001	p < 0.00001	p < 0.00001	
	**SIRS**				
Survivors, n = 100	1 (0–1)	2 (1–2)	1 (1–2)	1 (1–2)	p = 0.712
Non survivors, n = 12	2 (2–2)	2 (2–3)	2 (2–3)	2 (2–3)	p = 0.908
	p = 0.4354	p = 0.0214	p = 0.006	p = 0.006

**Fig. 2:**
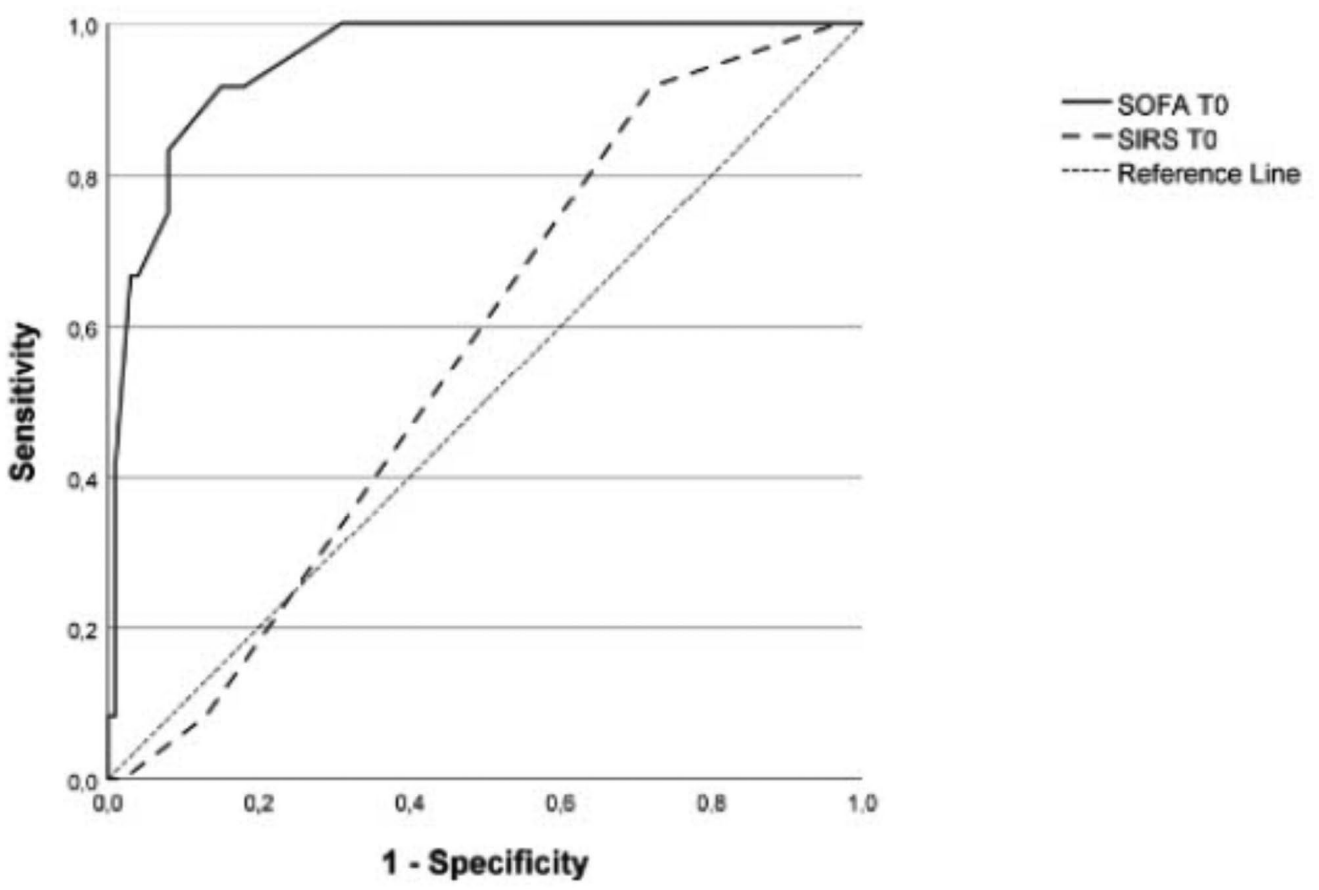
ROC curves for T_0_ nSOFA score and T_0_ SIRS criteria

ROC curve for T_0_ nSOFA showed AUC of 0.950 (95% C.I. 0.903–0.997) while ROC curve for T_0_ SIRS criteria showed AUC of 0.569 (95% C.I. 0.426–0.713); AUC was significantly higher for T_0_ nSOFA than T_0_ SIRS criteria (p = 0.0002) (Fig. 2). The best cut-off for T_0_ nSOFA was 4, with sensitivity 92% and specificity 85%.

In multivariate analysis including gestational age, Gram negatives, T_0_ and T_1_ nSOFA, and T_1_ SIRS criteria, T_0_ and T_1_ nSOFA remained significantly associated with mortality (p = 0.048 and p < 0.001, respectively) while T_1_ SIRS did not (Table 3). We decided not to include BW in the multivariate analysis model since it was collinear with gestational age. If birthweight was included in the model, T_0_ and T_1_ nSOFA remained significantly associated with mortality (p = 0.049 and p < 0.001, respectively) (Supplementary Table 1).

**Table 3:** Multivariate analysis model for the prediction of LOS-related mortality

Coefficients^a^								
Model		Unstandardized Coefficients		Standardized Coefficients	t	Sig	95,0% Confidence Interval for B	
		B	Std. Error	Beta			Lower Bound	Upper Bound
1	(Constant)	,061	,096		,634	,527	-,129	,250
	SOFA T_0_	-,024	,012	-,263	-2,003	,048	-,049	,000
	SOFA T_1_	,082	,012	,983	7,028	< ,001	,059	,105
	SIRS T_1_	,000	,029	,000	-,008	,994	-,057	,057
	Gram negatives	,053	,054	,067	,985	,327	-,054	,160
	Gestational age	,000	,000	-,091	-1,423	,158	,000	,000

^a^Dependent Variable: LOS-related death

## Discussion

Our study compared, for the first time, the prognostic accuracy of nSOFA score and SIRS criteria in predicting mortality in very preterm infants with LOS and we have demonstrated a greater discrimination capacity of nSOFA.

Neonatal SOFA was found to be higher in non-survivors vs. survivors at any time during the first 24 h from sepsis onset and to increase over time in non-survivors, while it did not vary in survivors. Moreover, multivariate analysis showed that both T_0_ and T_1_ nSOFA scores were independent predictors of mortality. These results confirm previous findings of higher nSOFA score in non-survivors vs. survivors during the first 48 h from onset of LOS in different cohorts of very preterm newborns [5, 20, 21]. The value of T_0_ nSOFA AUC (0.9498) indicates high accuracy for the prediction of LOS-related mortality, in agreement with the mean AUC of 0.88 reported in a previous multicenter study [5]. We found an optimal cut-off of 4 for T_0_ nSOFA to predict LOS-related mortality (sensitivity 92%, specificity 85%).

On the other hand, SIRS criteria did not discriminate between survivors and non-survivors at onset, although they were significantly higher in non-survivors vs. survivors at T_1_, T_2_ and T_3._ Lack of difference between survivors and non-survivors at T_0_ might be consistent with the diagnostic nature of SIRS criteria. At T_1_, T_2_ and T_3_ significantly higher SIRS criteria in non-survivors might be explained with persistence despite treatment, in comparison to survivors. However, in contrast to nSOFA score, SIRS criteria did not significantly increase over time in non-survivors, indicating poor association with unfavorable progression and outcome. Moreover, we found a sub-optimal AUC (0.5734) for T_0_ SIRS criteria and T_1_ SIRS criteria failed to predict mortality in multivariate analysis. On a whole, our findings show poor prognostic accuracy of SIRS criteria, partly attributable to the diagnostic nature of SIRS criteria. Globally, these results support the development and validation of specific scores for prognostic purposes.

The comparison of AUC of ROC curves showed significantly better discriminating capacity for T_0_ nSOFA vs. T_0_ SIRS criteria (p = 0.0002). Similarly, in septic patients admitted to PICU discrimination for in-hospital mortality was significantly higher for pSOFA than SIRS criteria, with AUC of 0.829 vs. 0.727 respectively (p < 0.01) [11] and in critically ill adults with suspected sepsis, an increase in SOFA score of 2 or more points showed a significantly higher discrimination for in-hospital mortality than the presence of at least 2 SIRS criteria [12]. A previous study showed the highest sensitivity of nSOFA occurring 24 and 48 h after onset and the highest specificity 6 h after onset [20], while higher AUC was found 12 h after onset in another study [21]. Despite these data, we decided to analyze T_0_ and T_1_ in order to evaluate the potential usefulness of nSOFA and SIRS criteria for early identification of high-risk patients during the course of LOS with the aim of prompting appropriate care in terms of monitoring and limiting organ impairment progression.

In multivariate analysis gestational age was preferred over birthweight because, from a pathophysiological perspective, the immunologic dysfunction observed in preterm newborns and predisposing to LOS and LOS-related mortality is attributable to immaturity itself [22–24]. Moreover, no small for gestational age infants, defined as birthweight < 3^rd^ centile for gestational age [25], was included among non-survivors, therefore our study could not detect the impact of such variable on LOS-related mortality. Finally, the inclusion of birthweight in the multivariate analysis did not significantly impact on the model.

Our findings highlight the pivotal importance of organ dysfunction assessment for the prognostic stratification of patients with sepsis as opposed to signs of inflammation. Our data are consistent with organ dysfunction progression demonstrated in newborns dying because of LOS, as oxygen requirement significantly increased from 3 days before death through the day of death, the need for mechanical ventilation and for vasopressors significantly increased from 2 days before death, while platelet count significantly decreased on the day before death [10].

In our population, non survivors presented lower gestational age and birthweight, and higher incidence of complications of prematurity, in comparison to survivors, in agreement with previous observations [1, 22], suggesting that baseline characteristics of patients might play a prevalent role in determining the outcome of LOS. However, according to multivariate analysis, for prognostic purposes, baseline characteristics as gestational age and birthweight are outperformed by scores of organ dysfunction.

Our study has some limitations. First, a relatively small number of non-survivors was included, and 4/12 patients died during the assessment period, causing one value for T_2_ and 3 values for T_3_ of nSOFA and SIRS criteria to be replaced by the maximal observed value for the patient. At present, no specific strategy to appropriately replace missing data in case of early death has been developed for studies on prognostic scores. Second, the criteria to establish the need for vasopressors or steroids with the purpose of maintaining blood pressure is still a matter of debate in newborns [26]. Patients in our cohort received medications for cardiovascular support basing on systemic blood pressure values and/or echocardiographic demonstration of abnormal cardiac function and low cardiac output according to local protocols. However, the monocentric design of our study was a strength, ensuring that the same local protocol was applied to all enrolled patients. Finally, because of the need to limit the number of samples in newborns for the hematologic component of SIRS and nSOFA after T_0_, relying on the latest values could be partially inaccurate.

In conclusion, our data indicate that nSOFA is an accurate prognostic tool for predicting mortality in preterm infants with LOS and shows higher discriminatory capacity for mortality than SIRS criteria. Hence, our findings discourage the use of SIRS criteria as prognostic scores and support the use of nSOFA score for prognostic stratification of preterm infants with suspected or proven LOS. Early identification of the subset of infants at greater risk of death is useful to plan patient-targeted management with the purpose of avoiding detrimental evolution of organ dysfunction and limiting LOS-related mortality.

### Supplementary Information

Below is the link to the electronic supplementary material.

Supplementary file1 (PDF 113 KB)

## Data Availability

The data that support the findings of this study are available from the corresponding author upon reasonable request.

## Abbreviations

AUC: Area Under Curve
CRP: C-reactive Protein
LOS: Late onset Sepsis
nSOFA: Neonatal Sequential Organ
PCT: Procalcitonin
pSOFA: Pediatric Sequential Organ Failure Assessment
PELOD: Pediatric Logistic Organ Dysfunction
ROC: Receiver Operating Characteristic Curve
SIRS: Systemic Inflammatory Response Syndrome
SOFA: Sequential Organ Failure Assessment
