# Supplementary material for: Prognostic accuracy of Neonatal SOFA score versus SIRS criteria in preterm infants with late-onset sepsis
Source: Eur J Pediatr. 2023 Aug 12;182(10):4731–9. doi: 10.1007/s00431-023-05143-5 (PMC10587306; doi:10.1007/s00431-023-05143-5)
Supplement: Supplementary file 1 — Supplementary file1 (PDF 113 KB) [file 431_2023_5143_MOESM1_ESM.pdf]

Supplementary Table 1 Multivariate analysis model for the prediction of LOS-related mortality, including birthweight.

Coefficients[a]

Model		Unstandardized Coefficients		Standardized Coefficients	t	Sig.	95,0% Confidence Interval for B	
		B	Std. Error	Beta			Lower Bound	Upper Bound
2	(Constant)	,297	,244		1,219	,226	-,186	,780
	SOFA T_0	-,024	,012	-,258	-1,961	,049	-,051	,000
	SOFA T_1	,081	,012	,966	6,870	<,001	,057	,104
	SIRS T_1	-,004	,029	-,008	-,130	,897	-,061	,054
	Gram negatives	,055	,054	,069	1,018	,311	-,052	,162
	Gestational age	,000	,000	-,065	-,960	,339	,000	,000
	Birthweight	-,010	,009	-,069	-1,055	,294	-,027	-,008

a. Dependent Variable: LOS-related death
